# A role of *BRCA1 *and *BRCA2 *germline mutations in breast cancer susceptibility within Sardinian population

**DOI:** 10.1186/1471-2407-9-245

**Published:** 2009-07-20

**Authors:** Grazia Palomba, Angela Loi, Antonella Uras, Patrizia Fancello, Giovanna Piras, Attilio Gabbas, Antonio Cossu, Mario Budroni, Antonio Contu, Francesco Tanda, Antonio Farris, Sandra Orrù, Carlo Floris, Marina Pisano, Mario Lovicu, Maria Cristina Santona, Gennaro Landriscina, Laura Crisponi, Giuseppe Palmieri, Maria Monne

**Affiliations:** 1 Istituto Chimica Biomolecolare-CNR, Trav. La Crucca – Baldinca Li Punti, 07100 Sassari, Italy; 2 Istituto di Neurogenetica e Neurofarmacologia-CNR, Cittadella Universitaria di Cagliari, 09042 Monserrato, Italy; 3 U. O. Ematologia, Ospedale San Francesco, Azienda USL3, via Mannironi, 08100 Nuoro, Italy; 4 Azienda USL1, Via Monte Grappa, 07100 Sassari, Italy; 5 Università di Sassari, Piazza Università, 07100 Sassari, Italy; 6 Ospedale Oncologico A. Businco, Azienda USL8, Via Jenner, 09100 Cagliari, Italy; 7 U.O. Oncologia, Ospedale C. Zonchello, Azienda USL3, via Mannironi, 08100 Nuoro, Italy

## Abstract

**Background:**

In recent years, numerous studies have assessed the prevalence of germline mutations in *BRCA1 *and *BRCA2 *genes in various cohorts. We here extensively investigated the prevalence and geographical distribution of *BRCA1-2 *mutations in the entire genetically-homogeneous Sardinian population. The occurrence of phenotypic characteristics which may be predictive for the presence of *BRCA1-2 *germline mutations was also evaluated.

**Methods:**

Three hundred and forty-eight breast cancer patients presenting a familial recurrence of invasive breast or ovarian carcinoma with at least two affected family members were screened for *BRCA1-2 *mutations by DHPLC analysis and DNA sequencing. Association of *BRCA1 *and *BRCA2 *mutational status with clinical and pathological parameters was evaluated by Pearson's Chi-Squared test.

**Results and Conclusion:**

Overall, 8 *BRCA1 *and 5 *BRCA2 *deleterious mutations were detected in 35/348 (10%) families; majority (23/35;66%) of mutations was found in *BRCA2 *gene. The geographical distribution of *BRCA1-2 *mutations was related to three specific large areas of Sardinia, reflecting its ancient history: *a*) the Northern area, linguistically different from the rest of the island (where a *BRCA2 c.8764_8765delAG *mutation with founder effect was predominant); *b*) the Middle area, land of the ancient Sardinian population (where *BRCA2 *mutations are still more common than *BRCA1 *mutations); and *c*) the South-Western area, with many Phoenician and Carthaginian locations (where *BRCA1 *mutations are prevalent). We also found that phenotypic features such as high tumor grading and lack of expression of estrogen/progesterone receptors together with age at diagnosis and presence of ovarian cancer in the family may be predictive for the presence of *BRCA1-2 *germline mutations.

## Background

Breast cancer is the most common malignancy in women in Western countries, currently accounting for one third of all female cancers [1]. In Sardinia, breast cancer represents the principal death-causing malignancy, with an incidence similar to that observed in western countries [2]. Familial aggregation is thought to account for 5–10% of all breast cancer cases, and germline mutations in different genes involved in pathways critical to maintain the genomic integrity have accounted for less than 25% of the inherited breast cancer [3,4]. Part of this familial clustering shows autosomal dominant inheritance with high penetrance due to mutations in the *BRCA1 *(MIM 113705) and *BRCA2 *(MIM 600185) breast cancer genes [5,6]. Cumulative breast cancer risks by age 70 are estimated to be 65% for *BRCA1 *and 45% for *BRCA2 *mutation carriers. In addition, women with *BRCA *mutations are at significant risk to develop ovarian cancer and other malignancies [7].

Hundreds of mutations among breast/ovarian cancer families have been found in these two genes. According to the Breast Cancer Information Core (BIC) database [8,9], about two thirds of germline variants identified in *BRCA1 *and *BRCA2 *are unique; the remaining ones are recurrent founder mutations which have been described in different ethnic groups and populations. Therefore, the mutation spectrum and prevalence of mutation carriers with breast or ovarian cancer depends on the population studied, and displays considerable variation based on ethnic and geographical diversity. In Italy, 4–27% of the identified mutations recurred among apparently unrelated families, while a regional founder effect has been demonstrated for few mutations [10-13]. In Sardinia, contribution of *BRCA1-2 *mutations to breast cancer predisposition has been reported for populations from the Northern part of the island [14]. Sardinian population shows strong founder effects for several genetic diseases with some geographical differences within the island [15,16]; indeed, some genetic heterogeneity has been described within the coastal or proximal-to-coast regions, which have suffered repeated invasions over many years, in comparison to the most internal and isolated part of the island [17]. On this regard, the assessment of both spectrum and prevalence of *BRCA1 *and *BRCA2 *mutations across the entire island as well as their predictive significance in this population is needed. Nevertheless, assessment of the likelihood of carrying deleterious mutations in *BRCA1 *or *BRCA2 *genes among patients who present high-risk cancer evaluation clinics will provide more accurate guidance to women and families about considering genetic testing [7].

Here we report on the prevalence and geographical distribution of *BRCA1-2 *mutations within the entire Sardinian population. We also investigated the existence of any significant association between mutations in *BRCA1-2 *genes and phenotypic characteristics of breast cancer patients that could drive specific treatment and influence the process of mutation testing.

## Methods

### Family selection

A total of 1,085 unselected breast cancer patients was recruited from all clinics at the Universities and Hospitals across the Sardinia island. To avoid any bias, patients were consecutively collected from January 1998 to December 2003 and were enrolled regardless of age at diagnosis, family history status or disease features. Sardinian origin was ascertained in all cases through genealogical studies. Family history of cancer was evaluated through specific questionnaires during the follow-up visits at the participating institutions. Three-hundred-forty-eight (32%) presented a familial recurrence of invasive breast or ovarian carcinoma: 237 originated from North Sardinia, 66 from Middle and 45 from South. Part of the present cohort (112 familial cases with at least three affected members) had been already included in our previous studies [18,19]. The remaining 737 (68%) breast cancer patients were sporadic cases.

Among patients with a family history of breast/ovarian cancer, 131/348 (37.6%) fulfilled the selection criterion for "high risk" families: presence of a breast cancer patient diagnosed before age 50 years and having at least one first- or second-degree relative diagnosed with breast cancer before 50 years of age or ovarian cancer at any age.

Of those, 73 originated from North Sardinia, 21 from the Middle and 37 from the South. The 81% of "high risk" families presented with breast cancer and the 12% with both breast and ovarian cancer. Male breast cancer was present in the 7% of the families (Table 1). The remaining 217 families were categorized as "no high risk" families: presence of a breast cancer patient having at least one first- or second-degree relative who is diagnosed with breast cancer or ovarian cancer at any age.

**Table 1:** *BRCA1 *and *BRCA2 *mutational analysis

Characteristics	Cases		*BRCA *positive Families	
	No.	%	No.	%
TOTAL CASES*	348		35	10

**"High risk"**	**131**		**23**	**17.5**
BC	106	81	17	16.0
BC/OC	16	12	5	31.2
MBC	9	7	1	11.1
Age at diagnosis <40 y	41	31	12	29.2
				
**"No High risk"**	**217**		**12**	**5.5**
BC	204	94	11	5.4
BC/OC	8	4	1	12.5
MBC	5	2	0	0.0
				
**2 BC/OC in the family**	**114**		**4**	**3.5**
≥ **3 BC/OC in the family**	**145**		**17**	**11.7**

**High risk**: presence of a breast cancer patient diagnosed before age 50 years and having at least one first- or second-degree relative who is diagnosed with breast cancer before 50 years of age or ovarian cancer at any age). **No high risk**: presence of a breast cancer patient having at least one first- or second-degree relative who is diagnosed with breast cancer or ovarian cancer at any age. BC, breast cancer; OC, ovarian cancer; MBC, male breast cancer.

* including 112 families already reported in our previous studies

According to number of breast/ovarian cancer in the families, we had 75 families with 3 or more affected members and 47 families with two affected members among the "high risk" cases; 67 families had 3 or more affected members and 70 families had two affected members among the "no high risk" cases. Information on total number of affected family members were uncertain or not available for 9 and 80 patients in the "high risk" and "no high risk" groups, respectively.

Clinical and pathological characteristics of the breast cancer probands including age at diagnosis, mono- or bilateral tumour location, stage of disease, tumour-node-metastasis system status, tumour grading, estrogen/progesterone receptor status, were confirmed by medical records and pathology reports.

Patients were informed about the aims of the study; blood samples for genetic testing were drawn after obtaining their written consent. The study was reviewed and approved by the ethical review boards of the participating Institutions accounting for samples' collection (University of Sassari; Azienda Sanitaria Locale of Sassari, Nuoro and Cagliari).

### Mutation detection

Mutation analysis of coding sequences and intron-exon boundaries of the *BRCA1 *and *BRCA2 *genes was carried out in the series of 348 probands from families with hereditary breast cancer. Genomic DNA was obtained from isolated white blood cells by salting out procedure [20]. Mutation detection for all coding regions and splice boundaries of *BRCA1 *and *BRCA2 *genes was performed by single strand conformation polymorphism (SSCP) for a small fraction of cases (27 family probands); all remaining cases underwent mutational screening by denaturing high performance liquid chromatography (DHPLC), using the WAVE Nucleic Acid Fragment Analysis System (Transgenomic, Omaha, NE), followed by automated sequencing as previously reported [18,19]. To confirm that each germline variant detected by sequencing was a real mutation and not a polymorphism, 103 unrelated normal individuals (corresponding to 206 chromosomes), originating from the same geographical area and with no family history for breast cancer, were used as controls and screened for each gene variant identified. For sequence homology searches, National Center for Biotechnology Information Blast database http://www.ncbi.nlm.nih.gov/BLAST/ has been used [21]. All identified variants were verified for their existence and nomenclature into the BIC database http://research.nhgri.nih.gov/bic/ and the Human Gene Mutation Database http://www.hgmd.cf.ac.uk/ac/[22-24].

### Statistical Analysis

The Pearson's Chi-Squared test was used for assessing the existence of any correlation between occurrence of deleterious germline mutations in *BRCA1*, *BRCA2*, or either gene and probands' characteristics. The following variables and categories were defined and included in our analyses: presence of ovarian or male breast cancer in family, synchronous or asynchronous bilateral breast carcinoma, age at diagnosis, histological tumor grade as standardized by Elston and Ellis [25], disease stage (pathological primary tumor size, pathological nodal status, presence of metastases) according to TNM classification which describes the anatomical extended of the disease by Sobin et al [26], estrogen and progesterone receptor status. The exact coefficient for sample proportion analysis was calculated to determine all of the significant parameters (below the 0.05 level). Odds ratios of carrying *BRCA1-2 *mutations were estimated by the logistic regression model and reported with 95% confidence interval (95% CI). All analyses were performed with the statistical package SPSS/7.5 for Windows.

## Results

### Family screening for BRCA1 and BRCA2 mutations

Genomic DNA from 348 probands of breast-ovarian cancer families was screened for germline mutations in *BRCA1 *and *BRCA2 *genes. Overall, deleterious germline mutations were detected in 35/348 (10%) familial cases: 23/131 (17.5%) among "high-risk" and 12/217 (5.5%) among "no high-risk" families (Table 1). Families with 3 or more BC cases, including the 112 reported in our previous studies, showed a prevalence rate of 11.7% (17/145). Presence of ovarian cancer in family increased the occurrence of *BRCA1-2 *germline mutations in either the subset of "high-risk" families (5/16; 31%) or the group of "no high-risk" families (1/8; 12.5%). No similar variation of mutation rates was observed in families presenting a male member with breast cancer. Stratification of families according to number of breast-ovarian cancer cases showed that *BRCA1/2 *positivity was significantly higher in families with 3 or more affected members than those with only two cases, with frequencies of 11.7% and 3.5%, respectively. Among 41/131 "high-risk" probands diagnosed at age ≤40 years, 12 (29%) presented a *BRCA1-2 *germline mutation.

The overall mutation prevalence was 7% in North Sardinia and 17% and 18% in families from Middle and South Sardinia, respectively. *BRCA *positivity accounts for 11% (8/73), 38% (8/21) and 19% (7/37) in "high risk" families from these geographical areas (data not shown).

### Spectrum of BRCA1-2 mutations in Sardinia

Overall, 8 distinct *BRCA1 *and 5 distinct *BRCA2 *pathogenic mutations were found in 35 (10%) out of 348 breast-ovarian cancer families; 11 "high-risk" families were positive for *BRCA1 *and 12 for *BRCA2*, whereas 1 *BRCA1 *and 11 *BRCA2 *mutations were identified in "no high-risk" families. Therefore, majority (23/35; 66%) of such mutations was found in *BRCA2*, and 12/35 (34%) were in *BRCA1*. All mutations were absent in normal genomic DNA from 103 unrelated healthy individuals (corresponding to 206 control chromosomes) and were classified as disease-causing variants due to their predicted effects on proteins. In Table 2, all germline mutations detected in our series have been reported. The *BRCA1 c.916_917delTT *mutation was the most represented *BRCA1 *variant (three positive families out of 348 cases; 1%), while the variants *c.8764_8765delAG *and *c.3950_3952delTAGinsAT *were the most recurrent mutations in *BRCA2 *among patients with a positive family history [13/348 (4%) and 7/347 (2%), respectively]. These mutations showed similar frequencies in both the "high risk" and no "high risk" groups (6.8% vs 5%).

**Table 2:** *BRCA1 *and *BRCA2 *pathogenetic mutations^a^

N° of positive families	GENE	EXON	DNA LEVEL	PROTEIN LEVEL	Mutation type	BIC
1	BRCA1	5	c.300 T>G	**p.Cys61Gly**	missense	YES

3	BRCA1	11	c.916_917delTT	**p.Val266SerfsX19**	frameshift	YES

1	BRCA1	11	c.1099_1100delCA	**p.Thr327fsX1**	frameshift	YES

1	BRCA1	11	c.1499insA	**p.Phe461fsX18**	frameshift	YES

2	BRCA1	11	c.1632 A>T	**p.Lys505X**	non sense	NO

1	BRCA1	11	c.1638 A > T	**p.Arg507X**	non sense	NO

2	BRCA1	11	c.3823_3826delACAA	**p.Asn1235fsX27**	frameshift	NO

1	BRCA1	14	c.4575delA	**p.Ser1486fsX18**	frameshift	NO

7	BRCA2	11	c.3950_3952delTAGinsAT	**p.Phe1241fsX17**	frameshift	NO

1	BRCA2	11	c.6586 C>G	**p.Ser2120X**	non sense	NO

1	BRCA2	11	c.6023_6024delTA	**p.His1932X12**	frameshift	YES

13	BRCA2	20	c.8764_8765delAG	**p.2846X22**	frameshift	YES

1	BRCA2	18	c.8559+1G>T	**-**	splicing	YES

^a ^Consensus nomenclature of mutations according to den Dunnen and Antonarakis [22]. Mutations refer to the GenBank accession #*BRCA1*: no. U14680 and *BRCA2*: no. NM_000059).

As indicated in Table 2, 4 out of 8 *BRCA1 *mutations and 2 out of 5 *BRCA2 *mutations have not been previously reported in either the BIC database. http://research.nhgri.nih.gov/bic/ or in the Human Gene Mutation Database http://www.hgmd.cf.ac.uk/ac/[23,24]. Besides deleterious mutations, complete *BRCA1-2 *screening identified 32 missense mutations and 16 intronic variations: 21 already described polymorphisms, 14 variants of unknown clinical significance and 13 novel variants not reported in the BIC database (Table 3).

**Table 3:** Other genetic variants in *BRCA1 *and *BRCA2*

Gene	Exon	Nucleotide	Codon	Intron	Base change	Mutation type	Nomenclature	BIC citation/Clinical importance
**BRCA1**		561–34		7	T > C		IVS7-34 T>C	YES/no
		667–57		8	delT		IVS8-57 delT	YES/no
	11	2196	693		G > A	missense	D693N	YES/no
	11	2731	871		C > T	missense	P871L	YES/no
	11	3232	1038		A > G	missense	E1038G	YES/no
	11	3667	1183		A > G	missense	K1183R	YES/no
	15	4654	1512		G > T	missense	S1512I	YES/no
	16	4956	1613		A > G	missense	S1613G	YES/no
				16	A > G		IVS16-68 A>G	YES/no
				16	A > G		IVS16-92 A>G	YES/no
	11	1186	356		A > G	missense	Q356R	YES/unknown
	11	1822	568		C > T	missense	P568L	YES/unknown
	11	2596	826		C > A	missense	T826K	YES/unknown
	11	4158	1347		A > G	missense	R1347G	YES/unknown
	11	4173	1352		G > A	missense	E1352K	YES/unknown
	16	5075	1652		G > A	missense	M1652I	YES/unknown
				17	C > T		IVS17-53 C>T	YES/unknown
				18	G > A		IVS18+65 G>A	YES/unknown
	5	287	56		A > T	missense	K56N	NO/unknown
	5	288	57		G > A	missense	G57R	NO/unknown
				17	G > T		IVS17-53 G>T	NO/unknown
				20	T > C		IVS20+34 T>C	NO/unknown
	22	5469	1784		G > A	missense	W1784I	NO/unknown

**BRCA2**	5'UTR	203			G > A	missense	203G>A	YES/no
	10	1379	384		C > T	missense	S384F	YES/no
	10	1093	289		A > C	missense	N289H	YES/no
	11	3199	991		A > G	missense	N991D	YES/no
	11	4469	1414		C > T	missense	T1414M	YES/no
	11	4486	1420		G > T	missense	D1420Y	YES/no
				16	T > C		IVS16-14 T>C	YES/no
	20	8731	2835		T > C	missense	S2835P	YES/no
	22			21	T > C		IVS21-66 T>C	YES/no
	27	10204	3326		A > T	nonsense	K3326X	YES/no
	1	353	42		C > T	missense	Y42C	YES/unknown
	10	1409	394		A > C	missense	E394A	YES/unknown
	11	3623	1132		A > G	missense	K1132R	YES/unknown
	11	6328	2034		C > T	missense	R2034C	YES/unknown
	11	6359	2044		G > C	missense	G2044A	YES/unknown
	25			24	T > C		IVS24-16 T>C	YES/unknown
	10	1343	372		A > C	missense	W372N	NO/unknown
				2	G > A		IVS2+65 G>A	NO/unknown
				4	A > C		IVS4+67 A>C	NO/unknown
		1042	272		A > T	missense	N272I	NO/unknown
	11	5967	1910		A > T	missense	N1910I	NO/unknown
				14	C > T		IVS14+53 C>T	NO/unknown
	14	7863	2546		T > C	missense	S2546P	NO/unknown
	16	7975	2583		G > A	missense	D2583N	NO/unknown
				16	T > A		IVS16-32 T>A	NO/unknown

**Table 4:** Logistic regression analysis to identify predictors of *BRCA *mutations

Characteristics	No. of cases	BRCA+ carriers	OR (95% CI)	P
**Age of Onset**				
< 45 years	139	21 (15%)	1.8 (1.05–4.28)	0.046
> 45 years	209	14 (7%)		
**Risk criteria**				
high-risk family	131	23 (18%)	3.2 (1.51–6.67)	0.002
no high risk family	217	12 (6%)		
**Ovarian cancer in family**				
presence	23	6 (26%)	3.7 (1.44–9.40)	0.006
absence	244	27 (11%)		
**Bilaterality**				
presence	36	8 (22%)	2.4 (1.05–5.39)	0.038
absence	228	25 (11%)		
**T**				
T1	151	12 (8%)	2.23 (0.57–8.82)	0.251
T2	70	10 (14%)		
T3-4	26	4 (15%)		
**N**				
N-	126	15 (12%)	1.02 (0.39–2.67)	0.963
N+	112	13 (12%)		
**M**				
M0	201	20 (10%)	1.33 (0.15–11.7)	0.797
M1	24	3 (12%)		
**Tumor Grading**				
G1-2	171	9 (5%)	11.3 (3.38–37.6)	< 0.001
G3-4	33	12 (36%)		
**Estrogen Receptor (ER)**				
ER-	55	16 (29%)	0.18 (0.06–0.53)	0.002
ER+	102	10 (10%)		
**Progesteron Receptor (PR)**				
PR-	81	21 (26%)	0.22 (0.07–0.66)	0.007
PR+	67	6 (9%)		

**Figure 1:**
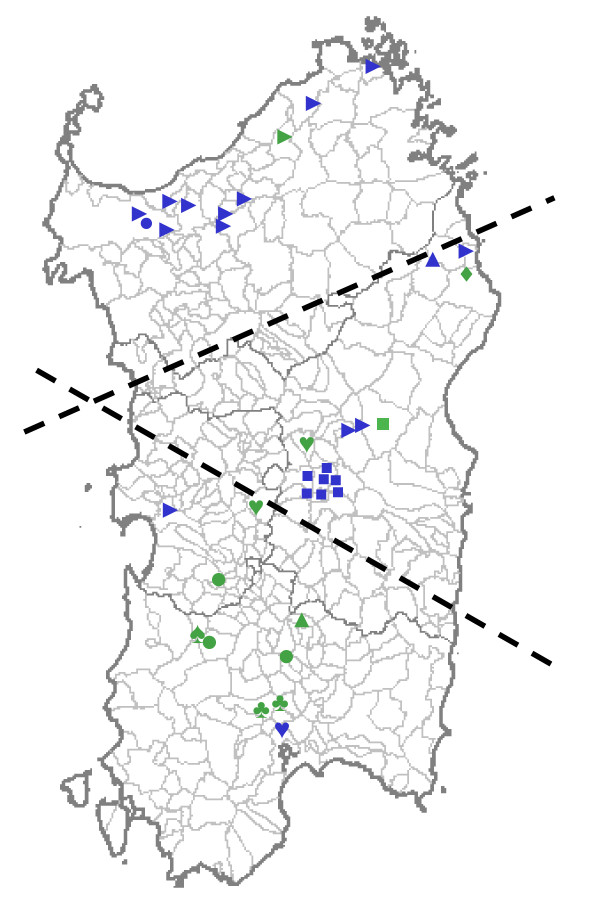
**Geographical distribution of BRCA1-2 mutations carriers in Sardinia**. Symbols indicate villages of origin for the patients presenting deleterious germline mutations in either *BRCA1 *or *BRCA2 *genes: "green square" *BRCA1 *c.300 T>G; green circle *BRCA1 *c.916_917delTT; "green triangle" *BRCA1 *c.1099_1100delCA; "green arrow" *BRCA1*c.1499insA; "green heart"*BRCA1 *c.1632 A>T; "green diamond" *BRCA1 *c.1638 A>T;"green clubs" *BRCA1 *c.3823_3826delACAA; "green spades" *BRCA1 *c.4575delA; "blue square" *BRCA2 *c.3950_3952delTAGinsAT; "blue triangle" *BRCA2 *c.6586 C>G; "blue circle" *BRCA2 c.6023_6024delTA*; "blue arrow" *BRCA2 *c.8764_8765delAG; "blue heart"*BRCA2 *c.8559+1G>T. Dashed lines delimit the three geographical regions reported in the text.

The geographical origin of the families positive for deleterious *BRCA1-2 *mutations is shown in Figure 1. The *BRCA1 *variants occurred in families originating from different areas of the island: 7 probands from the South, 2 from the Middle (*BRCA1 c.3823_3826delACAA, BRCA1 c.300 T>G*) and 1 from the North (*BRCA1 c.1632 A>T*). Carriers of the *BRCA1 c.916_917delTT *mutation were identified in three unrelated families from the South area. The *BRCA2 c.8764_8765delAG and BRCA2 c.3950_3952delTAGinsAT *variants were found in probands from the North and Middle Sardinia, whereas the remaining *BRCA2 *variants were detected in single families from the Central-East coast and South.

### Correlation between BRCA1-2 mutations and probands' phenotype characteristics

As shown in Table 4, univariate analysis indicated that presence of a *BRCA1-2 *mutation correlated significantly with earlier diagnosis age (P = 0.046), classification of the family as "high risk" (P = 0.002), recurrence in the family of ovarian cancer (P = 0.006), and development of a bilateral disease (P = 0.038). Considering the pathological parameters of primary tumors, no statistically significant correlation between *BRCA1-2 *mutations and primary tumor size (T) or axillary nodal status (N) or distant localization of the disease (M) was observed. However, the *BRCA1-2 *mutation rates were significantly higher in the subsets of patients with a more undifferentiated primary carcinoma (tumor grading 3–4, P < 0.001) and lack of expression of estrogen receptor (ER-, P = 0.002) or progesterone receptor (PR-, P = 0.007).

## Discussion

In this study, we have reported the prevalence of *BRCA1-2 *germline mutations in patients with a positive family history of breast and/or ovarian cancer from Sardinia, whose population shows genetic peculiarity due to geographical isolation and strong genetic drift [27,28]. Prevalence of *BRCA1-2 *mutations may indeed vary among distinct populations due to concurrence of different environmental factors and genetic backgrounds; in other words, patients origin may strongly account for different mutation rates in candidate genes.

In the present study, a germline pathogenic mutation in either *BRCA1 *or *BRCA2 *was identified in 10% of screened breast-ovarian cancer families (*BRCA1 *mutations were detected in about 3% of cases, while *BRCA2 *mutations were identified in about 7% of families). *BRCA *positivity reached 17.5% when considering the "high risk" families, 29% in families with probands diagnosed at age ≤40 years and 31% in the presence of ovarian cancer in the family. Prevalence rate in "high risk" families was higher than that of families with 3 or more affected member regardless of age at onset (11.7%). These results confirm that clinical characteristics such as ovarian cancer in the family, age at diagnosis and number of cases are good predictors for the likelihood to be a *BRCA *mutation carrier.

The overall prevalence of *BRCA1-2 *mutations among breast cancer patients from the entire Sardinia was quite similar to that previously reported for breast-ovarian cancer families originating from the Northern part of the island (for both studies, 15% *BRCA*-positive carriers were observed in breast-ovarian cancer families [14,18]. However prevalence of *BRCA *deleterious mutations was higher in Middle Sardinia (38% vs 11% in the North. and 19% in the South). When compared to many other Italian and European studies, the frequency is relatively low [10-13,29-32]. Since the sensitivity of mutation detection methods is not complete, some mutations may have remained undetected in the present study. Large genomic deletions, which do escape detection by both DHPLC and direct sequencing may account for a fraction of mutation-negative breast and ovarian cancer families in Sardinia. Overall and even considering a lack of sensitivity of the screening approach used in the present study, the prevalence of *BRCA *mutations in Sardinian families remains low. The selection criterion of two close relatives with breast cancer before the age of 50 years, used in this study, may somehow explain this low prevalence rate, though it is not possible to exclude that a specific genetic background may play a role on breast cancer susceptibility among Sardinian population. Noteworthy, a similar low prevalence of *BRCA *mutations was reported in the Finnish population which has genetic features comparable to the Sardinian one, where the historical, cultural and geographical isolation may have selected specific genetic variants as susceptibility genes for breast cancer [33].

The geographical origin of the families positive for deleterious *BRCA1-2 *mutations is shown in Figure 1. The *BRCA2 c.8764_8765delAG *and *c.3950_3952delTAGinsAT *variants were previously described as founder mutations in North and Middle Sardinia, respectively. In particular, cases carrying the *BRCA2 c.8764_8765delAG *mutation belonged to unrelated families originating from different villages in the northern part of the island [11,14]; most of families genotyped with markers flanking the *BRCA2 *gene at 13q12-q13 locus were demonstrated to share a large haplotype, not found in control chromosomes from the same geographical area [11]. Conversely, the *BRCA2 c.3950_3952delTAGinsAT*, which was previously reported as a founder mutation, was instead running in families belonging to a single extended pedigree confined to a small village of the central part of Sardinia [19]. The *BRCA1 *variants occurred in families originating from different areas of the island without a defined geographical clustering, though about half of the cases carrying the *BRCA1 *mutations originated from the South Sardinia. Interestingly, in three unrelated families from the South and Middle Sardinia we identified carriers of the *BRCA1 c.916_917delTT *mutation that has been reported in breast-ovarian cancer families from South Italy [34]. Altogether, the geographical distribution of the genetic variants in the island, suggests that *BRCA1-2 *mutations are related to the specific three large areas of Sardinia, which reflects its ancient history: a) the North area, delimited by the mountain chain crossing Sardinia and linguistically different from the rest of the island (where the *c.8764_8765delAG *variant acts as founder mutation); *b*) the Middle area, land of the ancient Sardinian population and domain of pastoral culture (where *BRCA2 *mutations are still more common than *BRCA1 *mutations); and *c*) the South-West area, with many Phoenician and Carthaginian archeological sites (where *BRCA1 *mutations are prevalent).

Majority of *BRCA1-2 *germline mutations identified in Sardinian families is unique: most of the variants were detected in single families and 6 were novel mutations.

The frequencies of *BRCA1 *or *BRCA2 *mutations among breast-ovarian cancer families varies widely between populations world-wide: Icelandic breast cancer families present almost exclusively *BRCA2 *mutations [30], roughly equal numbers of *BRCA1 *and *BRCA2 *mutations have been described in French Canadian or British breast cancer families [35,36], while a clear prevalence of *BRCA1 *mutations has been observed in the United States [37,38]. In this study *BRCA2 *mutations were more frequent than *BRCA1 *mutations, although it should be taken into account that a high proportion of families are carrying the *BRCA2 c.8764_8765delAG *founder mutation. Mutations in *BRCA2 *gene have been detected in up to 40% of male breast cancers in Iceland [39]; in our isolated population, we detected a *BRCA2 *mutation in 1/14 (7%) men with breast cancer with a frequency similar to that reported for male breast cancers in USA (4%) [40]. Again, these findings suggest that other environmental and/or genetic factors are contributing to the susceptibility to male breast cancer in Sardinia.

Specific pathological features have been reported in hereditary breast cancer with differences between *BRCA1 *and *BRCA2 *associated tumors [41]. In our series, the analysis of clinico-pathological characteristics showed some differences between *BRCA1-2 *wild type and mutation carriers. Though no correlation with the stage of disease (the main prognostic factor for breast cancer patients) was observed, the presence of *BRCA1-2 *mutations were significantly associated with some pathological characteristics (higher tumor grading, lack of expression of estrogen/progesterone receptors) which are recognized to have a negative impact on prognosis. Unfortunately, data from mutation screening of *BRCA1 *and *BRCA2 *were combined for the statistical analysis due to the low number of positive cases for each single gene in our series, although distinct differences in both molecular pathology and histopathology have been shown according to the specific carrier status.

## Conclusion

In summary, our results on the entire Sardinian population demonstrated that: *a*) prevalence of *BRCA1 *and *BRCA2 *mutations is 10% in familial breast-ovarian cancer; *b*) the spectrum of mutations among Sardinians is unique; *c*) some specific phenotypic features may be predictive for the presence of *BRCA1-2 *germline mutations and should be therefore considered when counselling Sardinian patients about undergoing genetic testing. On this regard, the fulfilment of one or more of the following criteria seems to increase the probability to detect a predisposing *BRCA1-2 *mutation among Sardinian breast cancer patients: *a*) high-risk classification of the patient's family; *b*) presence of at least one family member with ovarian cancer; *c*) occurrence of synchronous or asynchronous bilateral breast carcinoma.

Our findings further confirm that mutation frequency for *BRCA1 *and *BRCA2 *as cancer susceptibility genes for breast-ovarian carcinoma needs to be evaluated in each distinct geographical area. Finally, the absence of any *BRCA1 *or *BRCA2 *mutation in majority of "high-risk" families with breast-ovarian cancer support the general hypothesis that additional breast cancer susceptibility genes remain to be identified.

## Competing interests

The authors declare that they have no competing interests.

## Authors' contributions

GP, AL, AU, carried out the molecular genetic studies, participated in the sequence alignment and drafted the manuscript. PF, GP, MP, ML, participated to the DHPLC analysis and resequencing. AG, AC, AC, FT participated in the collection and assembly of clinical data. MB performed the statistical analysis. AF, SO, CF, MCS, GL participated in the provision of patients and revision of the histological records. LC participated in the design and coordination of the study, GP participated in the design and coordination of the study and helped to draft the manuscript, MM contributed to the study conception, obtained financial support and revised the manuscript. All authors read and approved the final manuscript.

## Pre-publication history

The pre-publication history for this paper can be accessed here:

http://www.biomedcentral.com/1471-2407/9/245/prepub
